# Enantioselective Modulatory Effects of Naringenin Enantiomers on the Expression Levels of *miR-17-3p* Involved in Endogenous Antioxidant Defenses

**DOI:** 10.3390/nu9030215

**Published:** 2017-02-28

**Authors:** Valeria Curti, Arianna Di Lorenzo, Daniela Rossi, Emanuela Martino, Enrica Capelli, Simona Collina, Maria Daglia

**Affiliations:** 1 Department of Drug Sciences, Medicinal Chemistry and Pharmaceutical Technology Section, Pavia University, Viale Taramelli 12, 27100 Pavia, Italy; valeria.curti86@hotmail.it or valeriacurti@kolinpharma.com (V.C.); arianna.dilorenzo01@universitadipavia.it or ariannadilorenzo@kolinpharma.com (A.D.L.); daniela.rossi@unipv.it (D.R.); simona.collina@unipv.it (S.C.); 2 KOLINPHARMA S.p.A., Lainate, Corso Europa 5, 20020 Lainate, Italy; 3 Department of Earth and Environmental Sciences, University of Pavia, Via S. Epifanio 14, 27100 Pavia, Italy; emanuela.martino@unipv.it (E.M.); enrica.capelli@unipv.it (E.C.)

**Keywords:** naringenin racemate, naringenin enantiomers, antioxidant enzymes, pro-inflammatory cytokines, caco-2 cells, microRNA, epigenetics

## Abstract

Naringenin is a flavanone present in citrus fruit as a mixture of chiral isomers. The numerous biological properties attributed to this compound include antioxidant and anti-inflammatory activities, even though the molecular mechanisms of these remain unknown. This study aims to evaluate the effects of racemic and enantiomeric naringenin on the expression levels of *miR-17-3p*, *miR-25-5p* and relative mRNA targets, to elucidate the mechanisms underlying these antioxidant and anti-inflammatory properties. Caco-2 cells, a well characterized in vitro model which mimics the intestinal barrier, were treated with subtoxic concentrations of racemate and enantiomers. The expression levels of *miR-17-3p* and *miR-25-5p* were determined by Real-Time PCR and were found to be decreased for both miRNAs. *miR-17-3p* behavior was in agreement with the increased levels of target mRNAs coding for two antioxidant enzymes, manganese-dependent superoxide dismutase (MnSOD) and glutathione peroxidase 2 (GPx2), while expression levels of *miR-25-5p* were not in agreement with its target mRNAs, coding for two pro-inflammatory cytokines, Tumor necrosis factor-alpha (TNF-α) and Interleukin-6 (IL-6). These results lead to the conclusion that naringenin could exert its antioxidant activity through epigenetic regulation operated by miRNAs, while anti-inflammatory activity is regulated by other miRNAs and/or mechanisms.

## 1. Introduction

Naringenin (5,7,4-thihydroxyflavanone) is a chiral flavonoid belonging to the class of flavanones. It is widely distributed in fruits of *Citrus* species, especially in grapefruit (*Citrus paradisi* Macfad.) and oranges (*Citrus* sinensis (L.) Osbeck), as well as tomatoes (*Solanum lycopersicum* L.) [1,2]. In *Citrus* fruits, naringenin is mainly found bound to glucose (naringenin-7-*O*-glucoside, also called prunin) [3], rutinose (naringenin-7-*O*-rutinoside, narirutin), and rhamnose (naringenin 7-rhamnoglucoside, naringin) [4]. In grapefruit, naringin content depends on the variety and ranges from 115 to 384 mg/L [5]. In tomatoes, naringenin is mainly found in its free form (ranging from 0.8 to 4.2 mg/100 g of whole red tomato) [2]. When naringenin is present as an aglycone, it occurs as a mixture of enantiomers whose ratio depends on the ripeness of the fruit and the purification methods used to isolate the isomers [6,7].

A number of in vitro studies have shown that naringenin possesses many physiological and pharmacological activities such as antioxidant and anti-inflammatory activities, which have been most studied, and hepatoprotective, anti-mutagenic and anticancer effects [1,6,8,9]. Thus, its bioavailability must be seen as a key aspect affecting its subsequent in vivobiological activity. Many studies have shown the presence of naringenin in both urine and in plasma following the intake of naringin, either alone or as part of *Citrus* fruit juices [10,11,12]. Despite the high rate of absorption that occurs especially at gut level, the naringenin bioavailability is poor, and this may be due to the first-pass effect and hepatic metabolism [13,14,15]. 

Despite its poor bioavailability, in vivo studies have shown that naringenin possesses anti-inflammatory and antioxidant capacities. Chtourou et al., in 2016, showed that administration of naringenin at a dose of 50 mg/body weight in rats previously exposed for 90 days to a high cholesterol diet, led to the restoration of both enzymatic and non-enzymatic antioxidant defenses (i.e., superoxide dismutase (SOD), catalase (CAT), glutathione peroxidase (GPx), nonprotein sulfhydryl groups (NPSH), glutathione (GSH), Vitamin C and Vitamin E) [16]. Ozkaya et al. showed that naringenin supplementation (50 mg/body weight) on the hepatic damage induced by lead (Pb) in rats increased CAT and GPx concentrations in groups of rats treated with naringenin and lead acetate, than in those treated with lead acetate alone [17]. Furthermore, Liu et al. have demonstrated that naringenin, administered at doses of both 25 and 50 mg/body weight/day, reduced oxidative stress in cardiorenal syndrome in a rat model. A similar increase was found in the mRNA levels coding for *Nrf2* (a well known transcription factor regulating antioxidant response), and *GCLc* (regulating the glutathione synthesis), underling that naringenin acts on transcription activity [18].

Moreover, recently, Chtourou et al. [19] showed that naringenin is able to decrease pro-inflammatory mediators such as *TNF-α*, *IL-6* and *IL-1b* in rats, and suggested that its anti-inflammatory activity could be due to the inhibition of NF-kB, a signal transduction pathway that promotes the transcription of gene coding for pro-inflammatory proteins. Recent studies have demonstrated that naringenin also possesses anti-inflammatory properties in the gut [20,21]. (*R*) naringenin was found to be the most effective enantiomer in reducing *TNF-α* and *IL-6* levels in human peripheral blood mononuclear cells (hPMBC). These results demonstrate that the anti-inflammatory effect of naringenin is enantioselective and that (*R*) naringenin is the eutomer [7].

Many studies have shown that polyphenols also modify gene expression by acting on the regulation of epigenetic mechanisms. They are able to modulate DNA methylation, hystonic modifications and microRNA (miRNA) expression [22]. miRNAs are small non-coding RNAs with an average length of 22 nucleotides, which control various biological processes such as cell development, differentiation, proliferation and apoptosis [23,24,25,26,27,28,29]. Literature data show that *miR-17-3p* and *miR-25-5p* are involved in the oxidative stress and inflammatory response, respectively. *miR-17-3p* has three mRNA targets coding for mitochondrial antioxidant enzymes (manganese superoxide dismutase (MnSOD), glutathione peroxidase-2 (GPx2), and thioredoxin reductase-2 (TrxR2)) [30]. *miR-17-3p* has been found to be regulated by different polyphenols, such as resveratrol and quercetin [31,32]. Our previous studies, which involved treating ECV-304 (human vascular endothelial cells) and hPBMC cells with increasing subtoxic concentrations of methyl 3-*O*-methyl gallate, demonstrated that this polyphenol induces an underexpression of *miR-17-3p*, and relative upregulation of target mRNA expression levels [33], placing this molecular mechanism at the base of the antioxidant effects registered in both in vitro and in vivostudies [34]. As far as *miR-25-5p* is concerned, it is strictly involved in the inflammatory response and its over or down regulation is associated with changes in cytokine levels during the inflammation process [35,36]. Few articles have been published on the capacity of naringenin to modulate miRNA expression to date. In 2012, Milenkovic et al. [37] reported that nutritional doses of naringenin resulted in a modification of miRNA expression levels in mouse liver. Taken together, these results make naringenin and its enantiomers promising candidates in risk prevention against the occurrence of inflammatory-based diseases, such as inflammatory bowel diseases.

Thus, considering: (a) the bioavailability of naringenin, albeit modest; (b) its involvement in the modulation of oxidative stress and inflammatory response (especially at the intestinal level); (c) the different activities exerted by naringenin enantiomers; and (d) its capacity to modulate the expression of some miRNAs, the aim of this investigation was to study the molecular mechanisms underlying the effects of racemic and enantiomeric naringenin on oxidative stress and the anti-inflammatory response, in which *miR-17-3p* and *miR-25-5p* play a key role.

## 2. Materials and Methods

### 2.1. Reagents and Instruments

(*R/S*) Naringenin (5,7-dihydroxy-2-(4-hydroxyphenyl)chroman-4-one) was obtained from Sigma-Aldrich (Milan, Italy). The HPLC grade solvents used as eluents were obtained from Merck-VWR (Milan, Italy). Analytical chiral resolutions were performed with a Jasco system consisting of a AS-2055 plus autosampler, a PU-2089 plus pump and a MD-2010 plus multi-wavelength detector coupled with a CD-2095 plus circular dichroism detector (Jasco Europe S.r.l., Cremella, LC, Italy). The preparative separations were carried out on an HPLC apparatus produced by Varian Chromatographic Systems (Walnut Creek, CA, USA), which consists of two Rainin SD-1 pumps with 500 mL/min pump heads, a 410 Varian autosampler, a 320 Varian Prostar UV-detector and a 320 Varian fraction collection module. A DIP 1000 photoelectric polarimeter from Jasco (JASCO Europe, Cremella, LC, Italy) was used for [α] measurements that were recorded at room temperature using a 1 dm cell and a sodium lamp.

MiRNeasy Mini kit was purchased from Qiagen GmbH (Hilden, Germany). A Quant-it RNA HS was purchased from Invitrogen (Grand Island, NY, USA). A Brilliant III Ultra-Fast SYBR^®^ Green RT-PCR Master Mix was purchased from Agilent Technologies (Santa Clara, CA, USA).

### 2.2. Naringenin Enantioresolution

The chiral resolution of naringenin was performed via preparative enantioselective chromatography employing a Chiralpak^®^ AD column (500 mm × 50 mm I.D., *d_p_* = 20 μm), Chiral Technologies Europe (Illkirch, France) according to Gaggeri et al. [8].Enantiomeric resolution in g-scale was achieved, eluting with methanol at room temperature with a flow rate of 40 mL/min. The partitioning of the eluate was effected according to the UV profile (detection at 290 nm) and the analytical in-process control of collected fractions performed using a Chiralpak AD-H column (250 mm × 4.6 mm ID; *d_p_* = 5 μm) eluting with methanol (flow rate 1 mL/min, UV detector at 290 nm). Accordingly, the fractions containing the enantiomers were evaporated at 334 mbar and 40 °C and dried in a vacuum oven at 0.1 mbar and 25 °C, furnishing2.1 g of (−)-(*S*) naringenin, (${\left[\alpha \right]}_{D}^{25}$ = −28.7°, *c* = 0.36% in ethanol, ee = 96%) and 1.8 g of (+)-(*R*) naringenin (${\left[\alpha \right]}_{D}^{25}$ = +22.8°, *c* = 0.30% in ethanol, ee = 94%).

### 2.3. Cell Culture and Treatments

Human colon adenocarcinoma (CaCo-2) cells were purchased from the American Type Culture Collection (Rockville, MD, USA). The cells were cultured in D-MEM supplemented with 10% fetal bovine serum, l-glutamine (2 mM), 100 IU/mL penicillin and 100 µg/mL streptomycin (all from Invitrogen Co., Paisley, Scotland, UK). Cells were grown at 37°C in a humidified atmosphere containing 5% CO_2_. The treatments were performed for 24 h with different concentrations of racemate and (*S*) and (*R*) enantiomers, with the concentration of DMSO never exceeding 0.4%. 

### 2.4. MTT Assay

Cell viability was detected by MTT assay, testing a wide range of doses of racemate and (*S*) and (*R*) naringenin enantiomers (i.e., from 1 to 1000 µg/mL). In brief, cells were seeded into permeable polyester membrane filter supports (Transwell, 12-mm diameter, 0.4-mm pore size; Corning Costar) at a density of 0.25 × 10^6^ cells/cm^2^. After a 24 h incubation period, the percentage of viable cells in each well was calculated relative to that of control cells.

### 2.5. RNA Extraction and Quantitative Real Time PCR (RT-PCR)

Total RNA was extracted from cells using the miRNeasy Mini kit (Qiagen, Hilden, Germany), according to the manufacturer’s instructions. The quality of RNA was assessed by gel electrophoresis using denaturing agarose gel 1.2%. Quantitative RNA analysis was performed using a fluorimetric method by means of the Qubit^R^ 2.0 platform (Invitrogen, Grand Island, NY, USA) using the Quant-iT RNA HS Assay with the following conditions: 2 µL of RNA was added to 198 µL of working solution obtained by mixing 1 µL of Qubit™ RNA HS reagent to 199 µL of Qubit™ RNA HSbuffer. Quantitative real-time PCR (RT-PCR) was done using cDNA obtained by a reverse transcription reaction using the miRCURY LNA™ Universal RT micro RNA PCR kit: 4 µL of total RNA (5 ng/µL) was added to 4 µL of 5× reaction buffer, 2 µL of enzyme mix, 1 µL of synthetic spike-in and 9 µL of nuclease free water. The mixture was then incubated in a thermo cycler (SureCycler 8800-Agilent Technologies, Santa Clara, California) at 42 °C for 60 min, 95 °C for 5 min, and then immediately cooled to 4 °C. To evaluate the expression of *miR-17-3p* and *25-5p*, RT-PCR reactions were performed with the AriaMX Real Time PCR System (Agilent Technologies, Santa Clara, California) using the Universal cDNA Synthesis and SYBRGreen Master Mix kits (Exiqon (Qiagen), Hilden, Germany). PCR amplification was performed in a 10 µL reaction mixture containing 4 µL of 1:80 diluted cDNA, 5 µL of SYBR Green master mix, and 1 µL of specific LNA probes (Exiqon, (Qiagen), Hilden, Germany) using the following reaction conditions: a first step at 95 °C for 10 min, 45 amplification cycles of 95 °C for 10 s followed by a step at 60 °C for 1 min. U6 small nuclear RNA (snU6) was used to normalize the expression data of miRNAs and every assay was performed in triplicate. To evaluate the levels of mRNA coding for MnSOD, GPx2 and TrxR2, which are validated targets of *miR-17-3p*, and of *TNF-a* and *IL-6*, which are targets of *miR-25-5p*,RT-PCR reactions were performed with the AriaMX Real Time PCR System using Brilliant III Ultra-Fast SYBR^®^ Green RT-PCR Master Mix (Agilent Santa Clara, California) according to the manufacturer’s protocol. Primers were designed using Primer-BLAST software (available online on 27 February 2017: http://www.ncbi.nlm.nih.gov/tools/primer-blast). The sequences for the used primers were:

MnSOD forward: 5′-AAACCTCAGCCCTAACGGTG-3′

MnSOD reverse: 5′-CCAGGCTTGATGCACATCTTA-3′

GPx2 forward: 5′-GAGGTGAATGGGCAGAACGA-3′

GPx2 reverse: 5′-CTCTGCAGTGAAGGGGACTG-3′

TNF-α forward: 5′-CCTCTCTGCCATCAAGAGCC-3′

TNF-α reverse: 5′-TTGAGTAACTTCGCCTGCGT-3′

IL-6 forward: 5′-GTCCAGTTGCCTTCTCCCTG-3′

IL-6 reverse: 5′-AGGGAATGAGGACACACCCA-3′

To determine relative mRNA expression, glyceraldehyde 3-phosphate dehydrogenase (GAPDH) was used as an endogenous control. The sequences for the used primers were:

GAPDH forward: 5′-CACTAGGCGCTCACTGTTCTC-3′

GAPDH reverse: 5′-GACTCCACGACGTACTCAGC-3′

### 2.6. Statistical Analysis

Statistical analysis of Cq values was carried out using software R (ver. 3.0.3, R e2sCore Team, 2014) [38]. Differences between group means were estimated using one-way analysis of variance followed by Tukey’s post hoc test, with measurements of *p* < 0.05 being taken as significant. In the figures, the mean ± standard deviation has been represented over repeats of inverse Delta Cq values (-Delta Cq) because these reflect the behavior of the expression levels of miRNA and mRNA directly.

## 3. Results

In order to dispose of both naringenin enantiomers, the resolution of commercially available (*R/S*) naringenin was accomplished via preparative enantioselective chromatography employing a Chiralpak AD-H column, as according to our previous protocol [7]. In brief, 5 g of (*R/S*) naringenin was processed, yielding (*S*) and (*R*) enantiomers of naringenin in quantities and purity suitable for an in depth biological investigation (chemical purity higher than 99%, ee > 94%).

**Figure 1:**
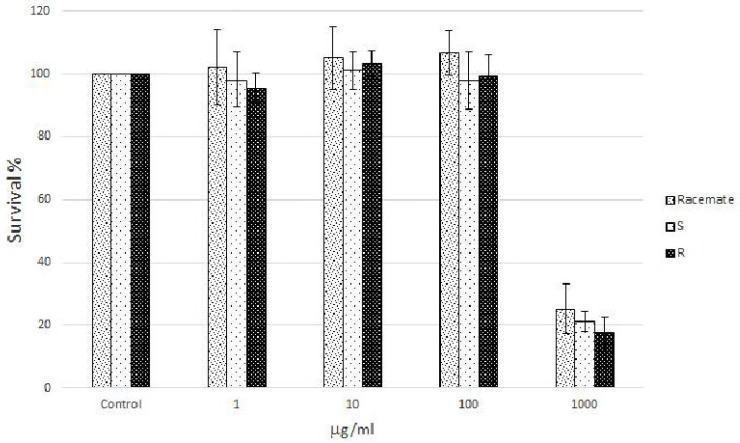
Survival percentage calculated against control (untreated cells) following the treatment of Caco-2 cells with increasing concentrations (1, 10, 100 and 1000 µg/mL) of racemic naringenin and, (*R*) and (*S*) enantiomers for 24 h. Boxes and error bars represent the mean ± standard deviation over repeat measurements.

Human Caco-2 cell cultures, a well characterized intestinal in vitro model which mimics the intestinal barrier and allows the evaluation of the effects of naringenin following oral consumption as a component of food, were treated with increasing concentrations (from 1 to 1000 µg/mL) of racemic and enantiomeric naringenin. At the highest concentration (1000 µg/mL), racemate and (*S*) and (*R*) naringenin enantiomers caused cell cytotoxicity rates of about 80%, while the lower concentrations (1, 10, and 100 µg/mL) were not cytotoxic (Figure 1). Thus, Caco-2 cell cultures were grown in the absence (control cell culture) and in the presence (treated cell cultures) of racemic and enantiomeric naringenin at concentrations of 1, 10, and 100 µg/mL.

**Figure 2:**
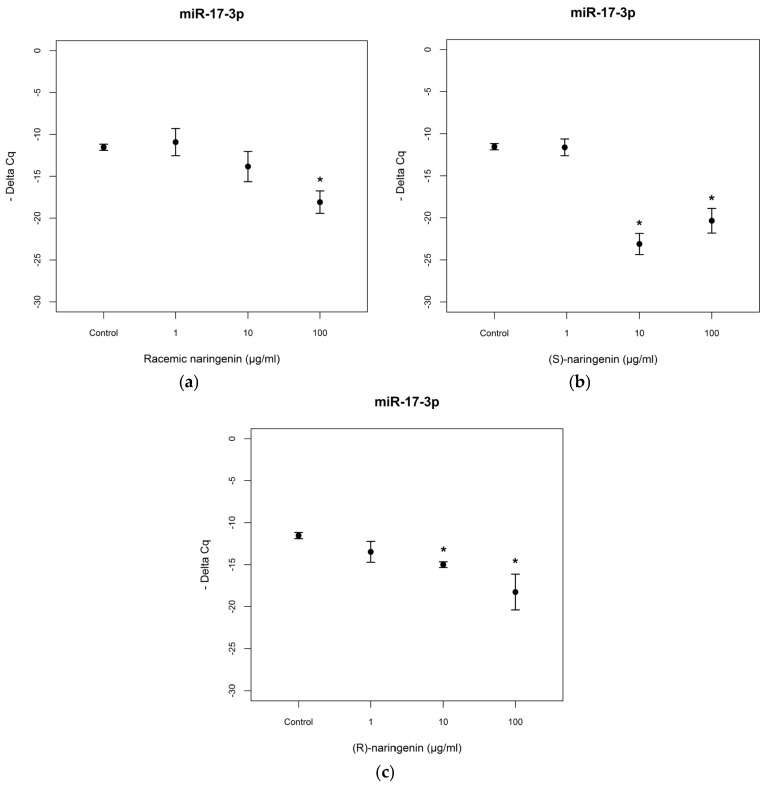
Expression levels, reported as Delta Cq, of miR-17-3p in Caco-2 cells treated with increasing concentrations of (**a**) racemic naringenin, (**b**) (*S*), and (**c**) (*R*) naringenin (1–100 µg/mL). * Indicates statistically significant differences between treated and untreated cell cultures, as reported in the text.

To evaluate the mechanisms through which naringenin exerts its antioxidant and anti-inflammatory activities, we determined the expression levels of *miR-17-3p* and *miR-25-5p* in Caco-2 cell cultures treated with racemic naringenin, (*S)* and (*R*) enantiomers. Total RNA was extracted from treated and control cell cultures, as according to the Material and Methods Section, and RT-PCR assays were performed. The results show that cell treatment with racemic naringenin at the highest concentration tested (100 µg/mL) induced a significant change (*F* = 9.459, *p* < 0.001) in the expression levels of *miR-17-3p*, leading to it being underexpressed (Tukey, *p* < 0.001). (*S*) and (*R*) naringenin enantiomers induced significant changes in *miR-17-3p* expression levels [(*S*) enantiomer: *F* = 28.173, *p* < 0.001; (*R*) enantiomer: *F* = 10.431, *p* < 0.001], leading to it being underexpressed at the higher concentrations tested, in comparison with untreated cell cultures [(*S*)enantiomer: Tukey, *p* < 0.001; (*R*) naringenin: Tukey, *p* < 0.01] (Figure 2). No statistical differences were registered (*p* > 0.05) between (*S*) and (*R*) enantiomer treatments.

**Figure 3:**
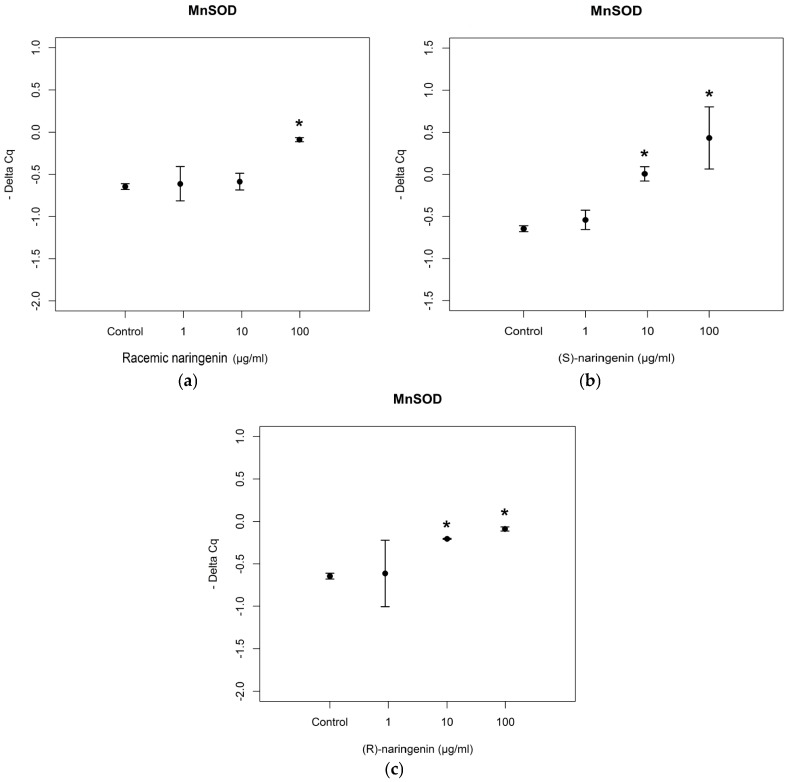
Expression levels, reported as Delta Cq, of mRNA coding for MnSOD in Caco-2 cells treated with increasing concentrations of (**a**) racemic naringenin, (**b**) (*S*), and (**c**) (*R*) naringenin (1–100 µg/mL). * Indicates statistically significant differences between treated and untreated cell cultures, as reported in the text.

Then, we investigated the expression levels of mRNAs coding for the antioxidant enzymes, MnSOD and GPx2, reported to be tagets of *miR-17-3p*. RT-PCR assays revealed that the racemate induced an overexpression of mRNA coding for MnSOD at the concentration of 100 µg/mL, in agreement with data obtained for *miR-17-3p* expression levels. Both enantiomers also induced an overexpression of mRNA coding for MnSOD at the higher concentrations tested [(*S*) enantiomer: *F*= 4.541, *p* < 0.05, Tukey, *p* < 0.05; (*R*) enantiomer: *F* = 105.65, *p* < 0.001, Tukey, *p* < 0.05] (Figure 3).

**Figure 4:**
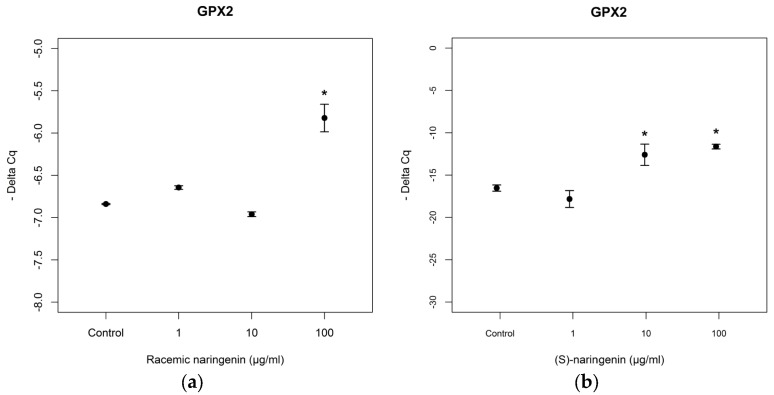

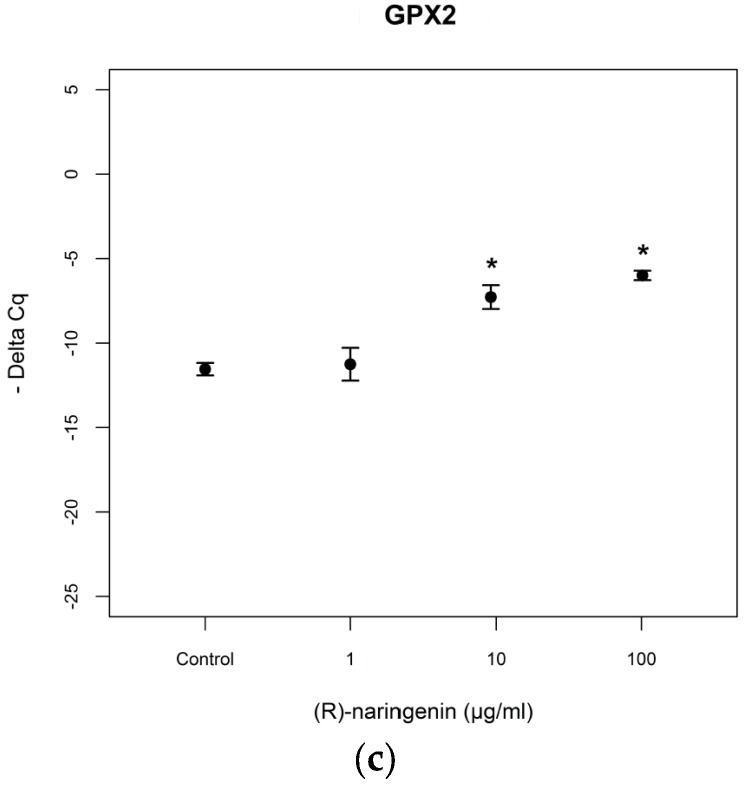
Expression levels, reported as Delta Cq, of mRNA coding for GPx2 in Caco-2 cells treated with increasing concentrations of (**a**) racemic naringin, (**b**) (*S*), and (**c**) (*R*) naringenin enantiomers (1–100 µg/mL). * Indicates statistically significant differences between treated and untreated cell cultures, as reported in the text.

As far as mRNA levels coding for GPx2 are concerned, the data obtained resulted to be similar to those obtained for mRNA coding for MnSOD (racemate: *F* = 75.97, *p* < 0.001, Tukey, *p* < 0.05; (*S*) naringenin: *F* = 14.94, *p* < 0.05, Tukey, *p* < 0.05; (*R*) naringenin: *F* = 7.89 *p* < 0.001, Tukey, *p* < 0.05) (Figure 4). No statistical differences (*p* > 0.05) were found between the effects of both enantiomers, on mRNA tagets of *mi-17-3p*.

**Figure 5:**
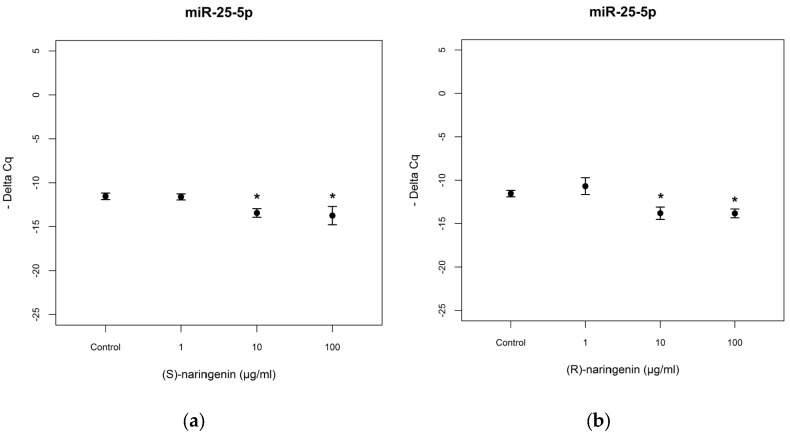
Expression levels, reported as Delta Cq, of *miR-25-5p* in Caco-2 cells treated with increasing concentrations of (**a**) (*S*), and (**b**) (*R*) naringenin enantiomers (1–100 µg/mL). * Indicates statistically significant differences between treated and untreated cell cultures, as reported in the text.

As far as *miR-25-5p* is concerned, the racemate did not induce significant miRNA variation (data not reported). In the cases of enantiomeric naringenin treatments, *miR-25-5p* levels decreased in cell cultures treated with 10 and 100 µg/mL for both enantiomers tested [(*S*) enantiomer: *F* = 3.544, *p* < 0.05; Tukey, *p* < 0.05; (*R*) enantiomer: *F* = 2.67, *p* < 0.05, Tukey, *p* < 0.05] (Figure 5). In this case too, no statistical differences were registered in the comparison of the treatments with the two enantiomers (*p* > 0.05).

**Figure 6:**
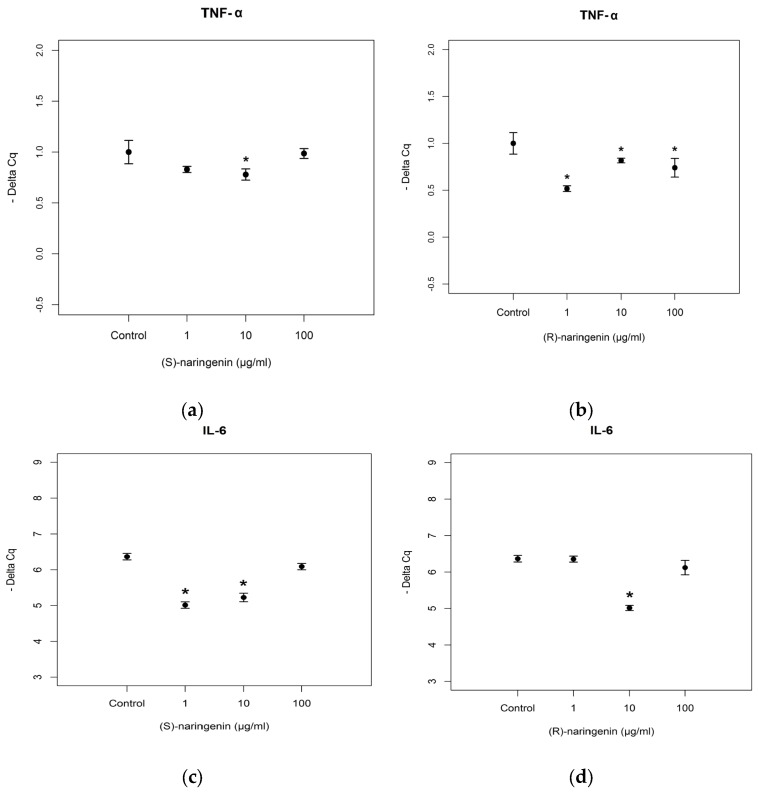
Expression levels, reported as Delta Cq, of mRNA coding for *TNF-α* in Caco-2 cells treated with increasing concentrations of (**a**) (*S*), and (**b**) (*R*) naringenin enantiomers (1–100 µg/mL) and and *IL-6* in Caco-2 cells treated with increasing concentrations of (**c**) (*S*) and (**d**)(*R*) naringenin enantiomers (1–100 µg/mL). * Indicates statistically significant differences between treated and untreated cell cultures, as reported in the text.

As far as mRNA targets are concerned, the expression levels of mRNAs coding for TNF-α and *IL-6* were studied. According to the results obtained for *miR-25-5p*, its mRNA targets did not show any variation in expression levels after treatment with racemate (data not reported). TNF-α mRNA expression levels were significantly decreased after (*S*)- and (*R*)-enantiomer treatments ((*S*)-enantiomer: *F* = 21.824, *p* < 0.05; (*R*)-enantiomer: *F* = 264.2, *p* < 0.001). For the (*S*)-enantiomer treatment, TNF-α mRNA was underexpressed in cells treated with 10 µg/mL (Tukey, *p* < 0.05), compared to control cell cultures. Treatment with (*R*)-enantiomer gave rise to TNF-α mRNA underexpression at all concentrations tested (Tukey, *p* < 0.05) including the lowest concentration (1 µg/mL). With regards to the expression levels of mRNA coding for IL-6, we registered underexpression following treatment with 1 and 10 µg/mL of the (*S*)-enantiomer, and with 10 µg/mL of the (*R*)-enantiomer ((*S*)-enantiomer: *F* = 44.591, *p* < 0.001, Tukey, *p* < 0.001; (*R*)-enantiomer: *F* = 27.945, *p* < 0.001; Tukey, *p* < 0.001) (Figure 6). No statistical differences (*p* > 0.05) were found between the effects of (*S)-* and (*R*)-enantiomer treatment on mRNA targets of *mi-25-5p*.

## 4. Discussion

Naringenin is a component of many plant foods commonly consumed within the diet. Therefore, this polyphenol can be considered a common constituent of our dietary pattern. Over the last decade, a large body of evidence suggests that naringenin exerts antioxidant and anti-inflammatory activities in in vitro and in vivostudies. Nevertheless, the mechanism of action of this flavanone is still largely unknown at a molecular level, as is the case for many other polyphenols. Thus, to unravel the potential mechanism of action of naringenin, we studied its effect on the expression levels of miRNAs involved in the endogenous antioxidant system (*miR-17-3p*) and inflammatory response (*miR-25-5p*) in human cells. In fact, these miRNAs are known to provide additional control to the complex regulation of gene expression at the post-transcriptional level, through the selective binding to complementary sequences of mRNAs coding for antioxidant enzymes and pro-inflammatory cytokines, besides other biochemical events ranging from extracellular stimuli, intracellular signaling pathways, and transcription factors. As far as the changes induced on *miR-17-3p* expression levels are concerned, in our experimental conditions, we found that (*S*) and (*R*) naringenin possessed similar activity, downregulating *miR-17-3p* from a concentration of 10 µg/mL. Racemate showed less activity, maintaining the ability to down-regulate *miR-17-3p* expression at only the highest concentration. These results seem to suggest that the enantiomers alone are more active than their equimolar mixture.

The biological significance of the modulation exerted by racemic and enantiomeric naringenin on the expression levels of *miR-17-3p* has been proven by the upregulation of the expression levels of the mRNAs coding for MnSOD and GPx2, which are reported to be validated targets of *miR-17-3p* [30]. In fact, in agreement with the downregulation of *miR-17-3p* levels, we registered an overexpression of mRNAs coding for MnSOD and GPx2 at identical concentrations of racemic and enantiomeric naringenin, respectively. In this case too, results obtained on mRNAs seem to suggest that the enantiomers alone, which showed similar activity, are more active than their equimolar mixture.

The fact that the expression levels of mRNAs coding for MnSOD and GPx2 are upregulated in response to the downregulation of *miR-17-3p* suggests a possible direct interaction between miRNA and naringenin that prevents the binding of *miR-17-3p* to its targets, allowing to these latter to be overexpressed. This hypothesis is in agreement with the results reported by Baselga-Escudero et al. that demonstrated the direct binding of some polyphenols to miRNAs by means of ^1^H NMR spectroscopy, suggesting a new posttranscriptional mechanism by which polyphenols could exert their modulation of protein synthesis. In addition, no statistical differences were evidenced for both(*S*) and (*R*) enantiomers, suggesting that the binding between miRNA and naringenin involves only the benzopyran moiety and not the chiral center [39].

As far as *miR-25-5p* is concerned, both enantiomers showed the capacity to modulate its expression, inducing the modification of the *miR-25-5p* expression levels only at the higher concentrations. Here too, a decrease of activity was registered in the presence of racemic naringenin, to the extent that no changes in the expression level of this miRNA are registered after this treatment. The expression levels of mRNAs coding for TNF-α and IL-6 are not in agreement with those obtained from *miR-25-5p*, as in both cases we have registered an under-expression. These results suggest that *miR-25-5p* is not the only miRNA involved in the modulation of TNF-α and IL-6 mRNA. In fact, literature data report that a single miRNA is able to bind hundreds of mRNAs, even if to date only a small fraction of miRNA–mRNA interactions has been validated experimentally [40]. These results show that naringenin enantiomers exert a beneficial effect regardless of their effect on the mRNA, inducing a reduction in the gene expression of mRNA coding for TNF-α and IL-6. 

Overall, the results obtained for these two latter mRNAs are consistent with those obtained from earlier studies performed in in vivoconditions on racemic naringenin and in in vitro conditions on naringenin enantiomers. In fact, Al-Rejaie et al. [41] showed that pretreatment with naringenin (50 and 100 mg/kg per day), or with mesalazine (300 mg/kg per day) used as positive control, for seven days before the induction of ulcerative colitis (through the treatment of 4% acetic acid), decreased the levels of pro-inflammatory cytokines (i.e., TNF-α, IL-1β, and IL-6) and prostaglandin E2. In addition, naringenin pretreatment induced an increase in catalase and superoxide dismutase at doses of 50 and 100 mg/Kg and 100 mg/kg, respectively confirming our data regarding the antioxidant activity. Moreover, regarding the in vitro studies, we demonstrated that in hPBMC cultures naringenin enantiomers significantly decreased pro-inflammatory cytokine levels (i.e., TNF-α and IL-6) [7].

## 5. Conclusions

This study confirms the epigenetic activity of racemic naringenin and reveals the effects of racemic and enantiomeric naringenin on the expression levels of *miR-17-3p*, involved in the antioxidant defense system, and *miR-25-5p*, involved in the anti-inflammatory response. As far as *miR-17-3p* is concerned, the downregulation of its expression levels correspond to an upregulation of mRNA coding for MnSOD and GPx2, showing an increase in antioxidant enzyme transcriptions and therefore in the antioxidant defense system. These findings can at least partly explain the well-known antioxidant activity of naringenin and, for the first time, show that this property is exerted through an epigenetic mechanism. As far as *miR-25-5p* is concerned, the underexpression of mRNA coding for the two cytokines, TNF-α and IL-6, is not supported by its behavior. This suggests that the modulation of these two mRNAs is not strictly under the control of *miR-25-5p*, and that this miRNA could act with other miRNAs, giving rise to synergistic and/or antagonistic effects.

Moreover, both naringeninenantiomers were found to be equally active on miRNAs at a higher level than the racemate, causing down-regulation in both cases, suggesting a negative synergism between enantiomers. (*S*) and (*R*) naringenin showed different activity on target mRNAs, suggesting that other epigenetic mechanisms (other miRNAs, DNA methylations, histone protein modifications) could be involved in the expression of the tested mRNAs. In addition, it seems to suggest that the mechanisms underlying these interactions, enantiomer–miRNA and enantiomer–mRNA, are different. Thus, further studies are needed to understand in what way naringenin and its enantiomers are able to modulate levels of miRNA, through both direct and indirect interaction. 

In conclusion, our results show the role of naringenin (both racemic and enantiomeric) as antioxidant agent acting through an epigenetic mechanism of action and support the hypothesis that long-term consumption of foods rich in naringenin could counteract oxidative stress through the increase of enzymatic antioxidant defense. The growing body of evidence demonstrating the antioxidant activity of naringenin as well asthese results lead to expect that naringenin could actually exert antioxidant effects also in vivo. Accordingly, in vivoinvestigations are needed for future applications of naringenin as an antioxidant agent in functional foods or food supplements.

## References

[1] Patel K., Singh G.K., Patel D.K. (2014). Review on pharmacological and analytical aspects of naringenin. Chin. J. Integr. Med..

[2] Bugianesi R., Catasta G., Spigno P., D’Uva A., Maiani G. (2002). Naringenin from cooked tomato paste is bioavailable in men. J. Nutr..

[3] Vallverdu’-Queralt A., Jauregui O., Medina-Remon A., Andres-Lacueva C, Lamuela-Raventos R.M. (2010). Improved characterization of tomato polyphenols using liquid chromatography/electrospray ionization linear ion trap quadrupole Orbitrap mass spectrometry and liquid chromatography/electrospray ionization tandem mass spectrometry. Rapid Commun. Mass Spectrom..

[4] Ho P.C., Saville D.J., Coville P.F., Wanwimolruk S. (2000). Content of *CYP3A4* inhibitors, naringin, naringenin and bergapten in grapefruit and grapefruit juice products. Pharm. Acta Helv..

[5] Tomas-Barberen F.A., Clifford M.N. (2000). Flavanones, chalcones and dihydrochalcones—Nature, occurrence and dietary burden. J. Sci. Food Agric..

[6] Orhan I.E., Nabavi S.F., Daglia M., Tenore G.C., Mansouri K., Nabavi S.M. (2015). Naringenin and atherosclerosis: A review of literature. Curr. Pharm. Biotechnol..

[7] Gaggeri R., Rossi D., Daglia M., Leoni F., Avanzini M.A., Mantelli M., Juza M., Collina S. (2013). An eco-friendly enantioselective access to (*R*)-naringenin as inhibitor of proinflammatory cytokine release. Chem. Biodivers..

[8] Gaggeri R., Rossi D., Christodoulou M.S., Passarella D., Leoni F., Azzolina O., Collina S. (2012). Chiral flavanones from AmygdaluslycioidesSpach: Structural elucidation and identification of TNFalpha inhibitors by bioactivity-guided fractionation. Molecules.

[9] Song H.M., Park G.H., Eo H.J., Lee J.W., Kim M.K., Lee J.R., Lee M.H., Koo J.S., Jeong J.B. (2015). Anti-Proliferative Effect of Naringenin through p38-Dependent Downregulation of Cyclin D1 in Human Colorectal Cancer Cells. Biomol. Ther. (Seoul).

[10] Wilcox L.J., Borradaile N.M., Huff M.W. (1999). Antiatherogenic properties of naringenin, a citrus flavonoid. Cardiovasc. Drug Rev..

[11] Erlund I., Meririnne E., Alfthan G., Aro A. (2001). Plasma kinetics and urinary excretion of the flavanones naringenin and hesperetin in humans after ingestion of orange juice and grapefruit juice. J. Nutr..

[12] Manach C., Morand C., Gil-Izquierdo A., Bouteloup-Demange C., Re’mesy C. (2003). Bioavailability in humans of the flavanones hesperidin and narirutin after the ingestion of two doses of orange juice. Eur. J. Clin. Nutr..

[13] Manach C., Scalbert A., Morand C., Rémésy C., Jiménez L. (2004). Polyphenols: Food sources and bioavailability. Am. J. Clin. Nutr..

[14] Felgines C., Texier O., Morand C., Manach C., Scalbert A., Régerat F., Rémésy C. (2000). Bioavailability of the flavanone naringenin and its glycosides in rats. Am. J. Physiol. Gastrointest. Liver Physiol..

[15] Orrego-Lagarón N., Martínez-Huélamo M., Vallverdú-Queralt A., Lamuela-Raventos R.M., Escribano-Ferrer E. (2015). High gastrointestinal permeability and local metabolism of naringenin: Influence of antibiotic treatment on absorption and metabolism. Br. J. Nutr..

[16] Chtourou Y., Kamoun Z., Zarrouk W., Kebieche M., Kallel C., Gdoura R., Fetoui H. (2016). Naringenin ameliorates renal and platelet purinergic signalling alterations in high-cholesterol fed rats through the suppression of ROS and NF-κB signaling pathways. Food Funct..

[17] Ozkaya A., Sahin Z., Dag U., Ozkaraca M. (2016). Effects of Naringenin on Oxidative Stress and Histopathological Changes in the Liver of Lead Acetate Administered Rats. J. Biochem. Mol. Toxicol..

[18] Liu Y., An W., Gao A. (2016). Protective effects of naringenin in cardiorenal syndrome. J. Surg. Res..

[19] Chtourou Y., Fetoui H., Jemai R., Ben Slima A., Makni M., Gdoura R. (2015). Naringenin reduces cholesterol-induced hepatic inflammation in rats by modulating matrix metalloproteinases-2, 9 via inhibition of nuclear factor κB pathway. Eur. J. Pharmacol..

[20] Azuma T., Shigeshiro M., Kodama M., Tanabe S., Suzuki T. (2013). Supplemental naringenin prevents intestinal barrier defects and inflammation in colitic mice. J. Nutr..

[21] Dou W., Zhang J., Sun A., Zhang E., Ding L., Mukherjee S., Wei X., Chou G., Wang Z.T., Mani S. (2013). Protective effect of naringenin against experimental colitis via suppression of Toll-like receptor 4/NF-κBsignalling. Br. J. Nutr..

[22] Blade C., Baselga-Escudero L., Arola-Arnal A. (2014). microRNAs as new targets of dietary polyphenols. Curr. Pharm. Biotechnol..

[23] Bartel D.P. (2004). MicroRNAs: Genomics, biogenesis, mechanism, and function. Cell.

[24] Wu X., Tan X., Fu S.W. (2015). May Circulating microRNAs be Gastric Cancer Diagnostic Biomarkers?. J. Cancer.

[25] Fu X.M., Zhou Y.Z., Cheng Z., Liao X.B., Zhou X.M. (2015). MicroRNAs: Novel Players in Aortic Aneurysm. BioMed Res. Int..

[26] Femminella G.D., Ferrara N., Rengo G. (2015). The emerging role of microRNAs in Alzheimer’s disease. Front. Physiol..

[27] Yanaihara N., Caplen N., Bowman E., Seike M., Kumamoto K., Yi M., Stephens R.M., Okamoto A., Yokota J., Tanaka T. (2006). Unique microRNA molecular profiles in lung cancer diagnosis and prognosis. Cancer Cell.

[28] Iorio M.V., Ferracin M., Liu C.G., Veronese A., Spizzo R., Sabbioni S., Magri E., Pedriali M., Fabbri M., Campiglio M. (2005). MicroRNA gene expression deregulation in human breastcancer. Cancer Res..

[29] Ozen M., Creighton C.J., Ozdemir M., Ittmann M. (2008). Widespread deregulation of microRNA expression in human prostate cancer. Oncogene.

[30] Xu Y., Fang F., Zhang J., Josson S., St Clair W.H., St Clair D.K. (2010). miR-17* suppresses tumorigenicity of prostate cancer by inhibiting mitochondrial antioxidant enzymes. PLoS ONE.

[31] Tili E., Michaille J.J., Alder H., Volinia S., Delmas D., Latruffe N., Croce C.M. (2010). Resveratrol modulates the levels of microRNAs targeting genes encoding tumor-suppressors and effectors of TGFβ signaling pathway in SW480 cells. Biochem. Pharmacol..

[32] Lesjak M., Hoque R., Balesaria S., Skinner V., Debnam E.S., Surjit K.S., Srai S.K., Sharp P.A. (2014). Quercetin inhibits intestinal iron absorption and ferroportin transporter expression in vivo and in vitro. PLoS ONE.

[33] Curti V., Capelli E., Boschi F., Nabavi S.F., Bongiorno A.I., Habtemariam S., Nabavi S.M., Daglia M. (2014). Modulation of human miR-17-3p expression by methyl 3-*O*-methyl gallate as explanation of its in vivo protective activities. Mol. Nutr. Food Res..

[34] Nabavi S.M., Habtemariam S., Nabavi S.F., Sureda A., Daglia M., Moghaddam A.H., Amani M.A. (2013). Protective effect of gallic acid isolated from Peltiphyllumpeltatum against sodium fluoride-induced oxidative stress in rat’s kidney. Mol. Cell. Biochem..

[35] Lee T.W., Tan E.L., Ng C.C., Gan S.Y. (2013). The Effect of Cytokines on MicroRNA Expression in TW01 Nasopharyngeal Carcinoma Cells. Br. J. Med. Med. Res..

[36] Mei Z., Chen S., Chen C., Xiao B., Li F., Wang Y., Tao Z. (2015). Interleukin-23 Facilitates Thyroid Cancer Cell Migration and Invasion by Inhibiting *SOCS4* Expression via MicroRNA-25. PLoS ONE.

[37] Milenkovic D., Deval C., Gouranton E., Landrier J.F., Scalbert A., Morand C., Mazur A. (2012). Modulation of miRNA expression by dietary polyphenols in apoE deficient mice: A new mechanism of the action of polyphenols. PLoS ONE.

[38] R Core Team (2014). R: A language and Environment for Statistical Computing, R Foundation for Statistical Computing, Vienna, Austria. http://www.R-project.org/.

[39] Baselga-Escudero L., Blade C., Ribas-Latre A., Casanova E., Suárez M., Torres J.L., Salvadó M.J., Arola L., Arola-Arnal A. (2014). Resveratrol and EGCG bind directly and distinctively to miR-33a and miR-122 and modulate divergently their levels in hepatic cells. Nucleic Acids Res..

[40] Helwak A., Kudla G., Dudnakova T., Tollervey D. (2013). Mapping the Human miRNA Interactome by CLASH Reveals Frequent Noncanonical Binding. Cell.

[41] Al-Rejaie S.S., Abuohashish H.M., Al-Enazi M.M., Al-Assaf A.H., Parmar M.Y., Ahmed M.M. (2013). Protectiveeffect of naringenin on acetic acid-induced ulcerative colitis in rats. World J. Gastroenterol..

